# Mechanistic Design in Photocatalysis

**DOI:** 10.1021/acs.accounts.5c00842

**Published:** 2026-03-05

**Authors:** Ronny Hardegger, Oliver S. Wenger

**Affiliations:** Department of Chemistry, 27209 University of Basel, St. Johanns-Ring 19, 4056 Basel, Switzerland

## Abstract

One of the most central questions in chemistry is how a starting material can be converted as simply and efficiently as possible into a product. The answer may include photocatalysis, and if the reaction proceeds well, one might argue that understanding the underlying mechanism is not essential. Even if the reaction does not perform as anticipated, condition screening may still provide the operationally simplest and most effective path to the desired outcome, while mechanistic aspects can remain largely unexamined. Given the large parameter space typically associated with modern photocatalytic reactions, this approach is both plausible and justified, particularly when product synthesis is the primary goal.

A complementary perspective on modern photocatalysis focuses on the conceptual advancement of photochemistry and a deeper understanding of its elementary steps and their interplay. This type of research begins with classical mechanistic elucidation to break down complex processes into individual elementary events. Once sufficient understanding has been achieved, it can lead to the mechanistic design of photoreactions. At that stage, the sequence of photophysical and chemical events triggered by light, and consequently the overall outcome of the reaction, can become rationally predictable, at least in principle.

In this Account, we examine how the cross-fertilization between synthetically oriented photoredox catalysis, which is primarily concerned with the activation and functionalization of organic molecules, and mechanistically driven research from the physical–inorganic domain has advanced the field of photochemistry. This interaction has often been catalyzed by controversial discussions surrounding the mechanistic details of reactions that have attracted significant synthetic interest. As a result, this interplay has propelled significant advances across several critical areas of modern molecular photocatalysis, including the reactivity of excited-state organic radicals and solvated electrons, the mechanisms underlying multiphoton excitation processes such as photon upconversion, the puzzling light-independent energy-loss phenomenon known as “cage escape”, and even the possibility of challenging Kasha’s rule, a foundational principle in photophysics with profound implications for photochemistry.

The knowledge accumulated through this work has brought the field closer to achieving mechanistically guided design in photocatalysis, extending far beyond the initial light-induced step. Central to this advancement are modern time-resolved spectroscopic methods, which have provided crucial insights into transient species and reaction dynamics. This conceptual strategy opens new opportunities and highlights challenges in redefining thermodynamic and kinetic limits. Ultimately, combining mechanistic insight with the practical expertise of synthetic chemists offers great potential for continued innovation in photoredox catalysis at the intersection of organic and physical–inorganic chemistry. With this Account, we aim to bridge the gap between those who prioritize the synthetic perspective and those who emphasize mechanistic and conceptual approaches, fostering greater integration between organic chemists and physical–inorganic chemists.

## Graphical abstract

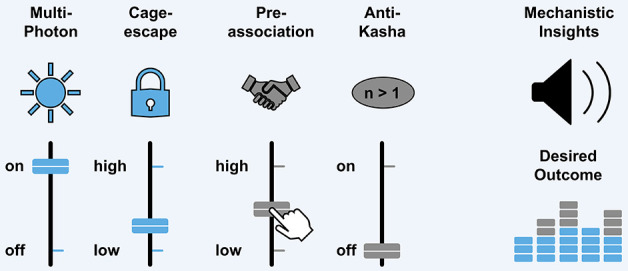

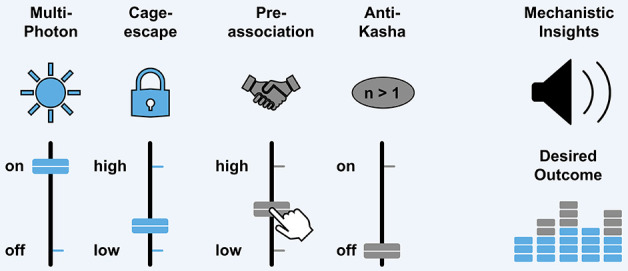

## Key References

-

Pfund, B. ; Wenger, O. S. Breaking Kasha’s Rule to Enable Higher Reactivity in Photoredox Catalysis. J. Am. Chem. Soc. 2025, 147, 26477–26485.40674569 10.1021/jacs.5c06115PMC12314908 ^1^ The dicyanoterphenyl radical anion has been observed to react directly from its second excited state with selected substrates, while its first excited state remains unreactive. This provides direct evidence of bimolecular photoreactivity that contradicts Kasha’s rule.
-

Pfund, B. ; Wenger, O. S. Picosecond reactions of excited radical ion super-reductants. Nat. Commun. 2024, 15, 4738.38834625 10.1038/s41467-024-49006-5PMC11150445 ^2^ After much debate over whether radical ions can drive photoredox catalysis, this work presents direct laser spectroscopic evidence supporting the idea, provided that ground-state preassociation with substrates enables picosecond electron transfer through excited-state quenching.
-

Wang, C. ; Bürgin, T. H. ; Li, H. ; Wenger, O. S. Cage escape governs photoredox reaction rates and quantum yields. Nat. Chem. 2024, 16, 1151–1159.38499849 10.1038/s41557-024-01482-4PMC11230909 ^3^ This study reveals that solvent cage escape is a key determinant in the efficiency of photoredox catalysis. We show that product formation rates in three benchmark reactions scale directly with the cage escape quantum yields of Ru^II^- and Cr^III^-based photocatalysts, providing mechanistic insight rooted in Marcus theory of electron transfer.
-

Glaser, F. ; Kerzig, C. ; Wenger, O. S. Sensitization-initiated electron transfer via upconversion. *Chem. Sci.* 2021, 12, 9922.10.1039/d1sc02085dPMC831764734349964 ^4^ This study contributed to resolving a controversy regarding the mechanism of a conceptually novel approach to photoredox catalysis.

## Introduction

Modern photochemistry is shaped by researchers from diverse backgrounds and different interests. For many practitioners, synthetic innovation is the main motivation, whereas for others it is the fundamental understanding of photochemical elementary reaction steps. Both perspectives are important and ideally complementary, and while some studies successfully address both aspects, in most cases one of the two tends to dominate a given piece of research. One key reason for this is that the knowledge and experimental settings required to drive organic synthetic innovation are typically quite different from those needed for the mechanistic elucidation of reactions, which may demand more physical-inorganic expertise and specialized spectroscopic equipment.^5,6^

Here, we attempt to bridge these two perspectives by focusing on mechanistic insights as the basis of mechanistic design, which we consider the ultimate goal of mechanistic elucidation in photochemistry: the ability to rationally design a complete photochemical reaction mechanism with a quantitative understanding of all involved elementary steps, leading to a predictable final product. This concept is not per se a distinct conceptual advance beyond long-standing goals in photochemistry, but it remains a useful organizing theme and an overarching goal for the research presented herein.

As is customary for articles in this journal, we focus on selected topics our group has engaged with over the past few years, set within the broader context of related work by other research groups, while respecting the format restrictions of this Account. We begin with a mechanistic controversy concerning the operating principle of a multiphoton excitation process that has helped assess the potential of photon upconversion in photoredox catalysis. We then show how luminescence quenching can be an unreliable indicator of successful photoreactivity, as it is followed by an often-overlooked light-independent elementary step known as cage escape, involving the separation and possible recombination of primary radical pair photoproducts. This brings us to a broader mechanistic debate: whether excited organic radicals can undergo photochemistry on the picosecond time scale, or whether degradation products and solvated electrons play a more significant role. Our identification of experimental conditions that support picosecond photochemistry of organic radicals leads us to the phenomenon of anti-Kasha behavior, in which a molecule in solution reacts directly from a higher electronically excited state, a highly unusual reaction pathway in bimolecular solution-phase chemistry. We conclude with remarks on the current status of mechanistic insights and perspectives on promising future directions.

## Photoredox Catalysis via Photon Upconversion

In 2017, the König group introduced the concept of sensitization-initiated electron transfer to preparative-scale photoredox catalysis.^7^ Upon excitation of [Ru­(bpy)_3_]^2+^ (bpy = 2,2′-bipyridine) in the presence of excess pyrene as a cocatalyst and diisopropylethylamine (DIPEA) as a reducing agent, the reductive dehalogenation of aryl halides was observed, leading to the formation of aryl radicals and, subsequently, stable C–H arylation products. The reaction was proposed to proceed via triplet–triplet energy transfer (TTET) from [Ru­(bpy)_3_]^2+^ to pyrene, followed by reductive quenching of triplet-excited pyrene to form the pyrenyl radical anion, which is thermodynamically competent for the reductive dehalogenation of the investigated substrates (Figure 1a). However, as noted by Ceroni and co-workers, a key issue with this mechanistic proposal is that electron transfer from DIPEA to triplet-excited pyrene is thermodynamically uphill by 0.9 eV and therefore implausible.^8^ König and co-workers responded that several mechanisms are conceivable and that spectroscopic studies aimed at elucidating reaction pathways often need to be conducted under idealized conditions, which may not exactly replicate those of preparative photoredox catalysis.^9^ While both points are reasonable, they did not seem entirely satisfactory to us and to others.^4,10^

**Figure 1:**
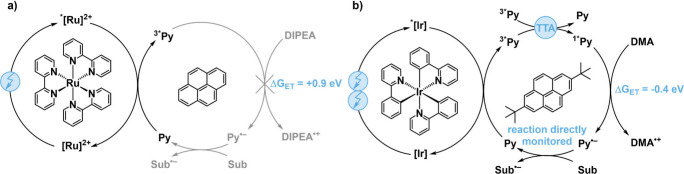
(a) Initially proposed mechanism for sensitization-initiated electron transfer, involving an endergonic (Δ*G*_ET_ = +0.9 eV) electron transfer from diisopropyl ethylamine (DIPEA) to triplet-excited pyrene (^3*^Py).^7^ (b) Refined mechanism incorporating triplet–triplet annihilation upconversion (TTA-UC) to generate singlet-excited pyrene (^1*^Py), enabling exergonic (Δ*G*_ET_ = −0.4 eV) electron transfer from *N*,*N*-dimethylaniline (DMA).^4^ The free energies for electron transfer reactions (Δ*G*_ET_) were estimated based on known redox potentials.^11^

Using a slightly different yet conceptually analogous photosensitizer and cocatalyst combination comprised of *fac*-[Ir­(ppy)_3_] (ppyH = 2-phenylpyridine) and 2,7-di-*tert*-butylpyrene (Figure 1b), we demonstrated that TTET from the metal complex to the pyrene is followed by triplet–triplet annihilation upconversion (TTA-UC).^4^ TTA-UC relies on the encounter of two triplet-excited molecules, generated by a biphotonic process, to form a higher-energy singlet excited state.^12^ Experimentally, the biphotonic nature of TTA-UC has been confirmed by the characteristic quadratic dependence of upconverted pyrene emission on excitation intensity. In the resulting S_1_ excited state, pyrene is thermodynamically competent to oxidize tertiary amines (including *N*,*N*-dimethylaniline (DMA)), as this S_1_ state stores approximately twice the energy of its T_1_ state. Furthermore, we directly observed the resulting pyrenyl radical anion by transient UV–visible absorption spectroscopy and monitored its reaction with the substrate. Such direct observation of a catalytically active species and its subsequent reaction is rare, but represents an ideal scenario. Our findings confirmed the hypothesis proposed by Ceroni in response to König’s original mechanistic interpretation, namely that a TTA-UC step could be involved.^8^

In separate independent studies, another team concluded that within the [Ru­(bpy)_3_]^2+^/pyrene/DIPEA system, there may be simultaneous formation of one-electron reduced ruthenium complexes, [Ru­(bpy)_3_]^+^, and triplet-excited pyrene.^10^ These two transient species are proposed to react and produce pyrenyl radical anions, although the latter were not directly observed. This mechanism is expected to depend strongly on the relative concentrations of the individual reaction components, as these determine the concentrations of [Ru­(bpy)_3_]^+^ and triplet-excited pyrene. This alternative pathway resembles mechanisms proposed for certain Birch-type photoreductions,^13^ which are likewise hypothesized to involve two short-lived intermediates.^14^ We believe there is significant potential for further mechanistic clarification and research in this context.

Photon upconversion has recently attracted significant attention from the photoredox community as a strategy to utilize the longest possible input wavelengths while still enabling thermodynamically demanding reactivity.^15^ Red-light-driven photoreactivity can be attractive, for example, in situations where shorter-wavelength light is absorbed by components that are not intended to be excited, where it lacks sufficient penetration depth, or where it causes photodamage. Interest in red-light-driven upconversion increased in 2019 with a significant publication that used Pd^II^ and Pt^II^ sensitizers together with annihilator molecules to enable photoreactions that typically require shorter-wavelength light.^16^ These reactions proceeded either directly from the singlet excited state (S_1_) of the annihilator or via energy transfer to a conventional cocatalyst such as Eosin Y or [Ru­(bpy)_3_]^2+^. In the latter strategy, one can distinguish between approaches where the upconversion system and the cocatalyst are in the same reaction mixture and those where they are confined to spatially separate compartments.^17^

In our own studies on red-light-driven photocatalysis, we found that the choice of red-light-absorbing sensitizer can determine whether the overall reaction proceeds through reductive or oxidative chemistry, due to a shift in the underlying mechanism. The [Os­(bpy)_3_]^2+^/DCA sensitizer–annihilator pair (DCA = 9,10-dicyanoanthracene) enables upconversion of red light followed by oxidative photochemistry from the singlet-excited state of DCA (Figure 2a).^19^ In contrast, the [Cu­(dap)_2_]^+^/DCA system (dap = 2,9-dianisyl-1,10-phenanthroline) appears to follow pathways that generate DCA radical anions and potentially further reduced degradation products,^18^ resulting in overall reductive chemistry (Figure 2b) in the presence of excess tertiary amines.^20^ This demonstrates a form of mechanistic control in photocatalysis through sensitizer selection.

**Figure 2:**
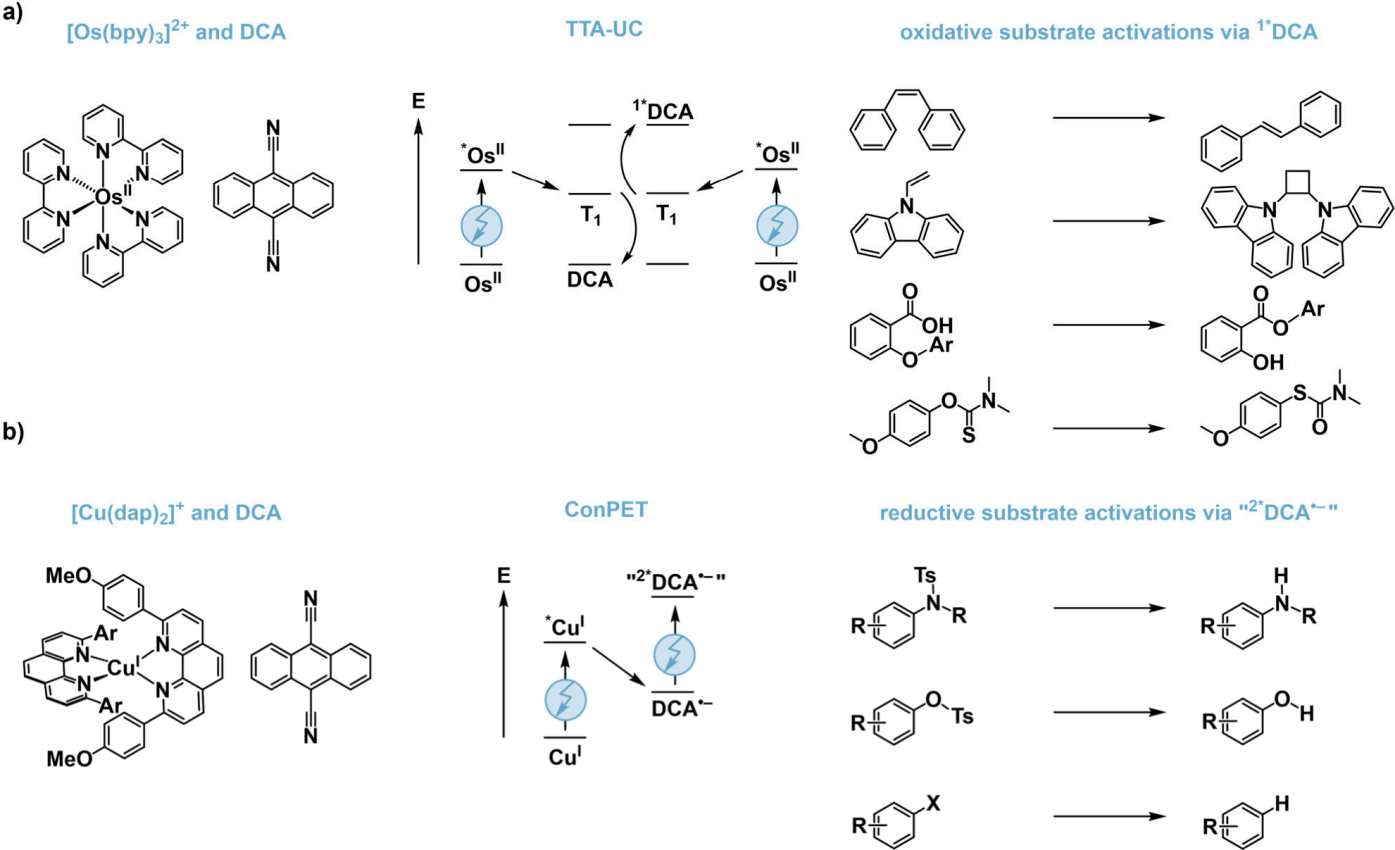
Sensitizer-controlled photoreactivity. (a) Combination of [Os­(bpy)_3_]^2+^ and DCA enabling red-to-blue triplet–triplet annihilation upconversion (TTA-UC), followed by oxidative chemistry from singlet-excited DCA.^19^ The illustrated photoisomerization, [2 + 2] photocyclization, and rearrangement reactions are redox-neutral yet are initiated by substrate oxidation through singlet-excited DCA. (b) Combination of [Cu­(dap)_2_]^+^ and DCA in the presence of a tertiary amine electron donor generating DCA radical anions and likely reducing degradation products. Further excitation with red light drives reductive chemistry.^20^

More recently, upconversion into the ultraviolet spectral region has become a key area of focus,^21^ as UV light sources are comparatively scarce, and considerations of photodamage and light penetration are especially relevant for the UV spectral range. Effective upconversion to the UV has been achieved using organic chromophores and iridium­(III) complexes as sensitizers,^22–24^ paired with acetylene-substituted biphenyl and benzene derivatives as annihilators,^25,26^ building on earlier strategies involving polyaromatic hydrocarbon annihilators.^27,28^ Upconversion reaching energies of approximately 4.3 eV in the UV–C region has been demonstrated with benzene derivatives.^23,25^ While UV upconversion so far proceeds mostly with very modest efficiencies, in selected cases efficiencies up to 20% have already been reported.^29^

This level of efficiency is particularly important when coupling upconversion with subsequent photochemical transformations.^30^ The situation becomes more straightforward when the annihilator can undergo a direct photoreaction after upconversion, such as the dimerization of anthracene molecules.^31^ However, follow-up photochemistry, that involves a substrate molecule rather than the annihilator, has been mostly limited to singlet-state processes and photodissociation reactions.^26,32^ Triplet follow-up reactions remain rare, as upconversion yields singlet excited annihilators, and generating high-energy triplet states on substrate molecules, after singlet–singlet energy transfer and intersystem crossing, introduces energy loss pathways that are difficult to control.^33^ The specific challenge is that once high-energy triplet states on substrate molecules are formed, these states can be quenched by the lower-energy triplet excited states of the excess annihilator molecules.

We conclude this section by noting that the studies summarized here demonstrate that biphotonic strategies, combined with detailed mechanistic understanding, provide a powerful means to broaden the design space for future photochemical transformations, although upconversion efficiencies may ultimately become a limiting factor.^100^

## Role of Cage Escape

Many photoredox studies conclude from luminescence quenching experiments that a successful photoinduced electron transfer (PET) reaction has occurred. This conclusion is only valid insofar as a primary radical pair composed of the photocatalyst and the substrate in altered redox states has been formed. However, this radical pair remains embedded in what is called the solvent cage. The two radical photoproducts must escape this cage for a productive onward reaction to become possible. Spontaneous back electron transfer competes effectively with cage escape and in the worst case leads to complete radical recombination and no productive onward reaction (Figure 3a), even though substantial luminescence quenching is observed. Many factors including solvent polarity and viscosity, temperature, reaction free energy for back electron transfer inside the cage, and spin effects influence the cage escape quantum yields.^34^ Predictions about cage escape quantum yields are therefore very difficult to make. Their experimental determination requires access to specialized equipment such as transient ultraviolet visible absorption spectroscopy.

**Figure 3:**
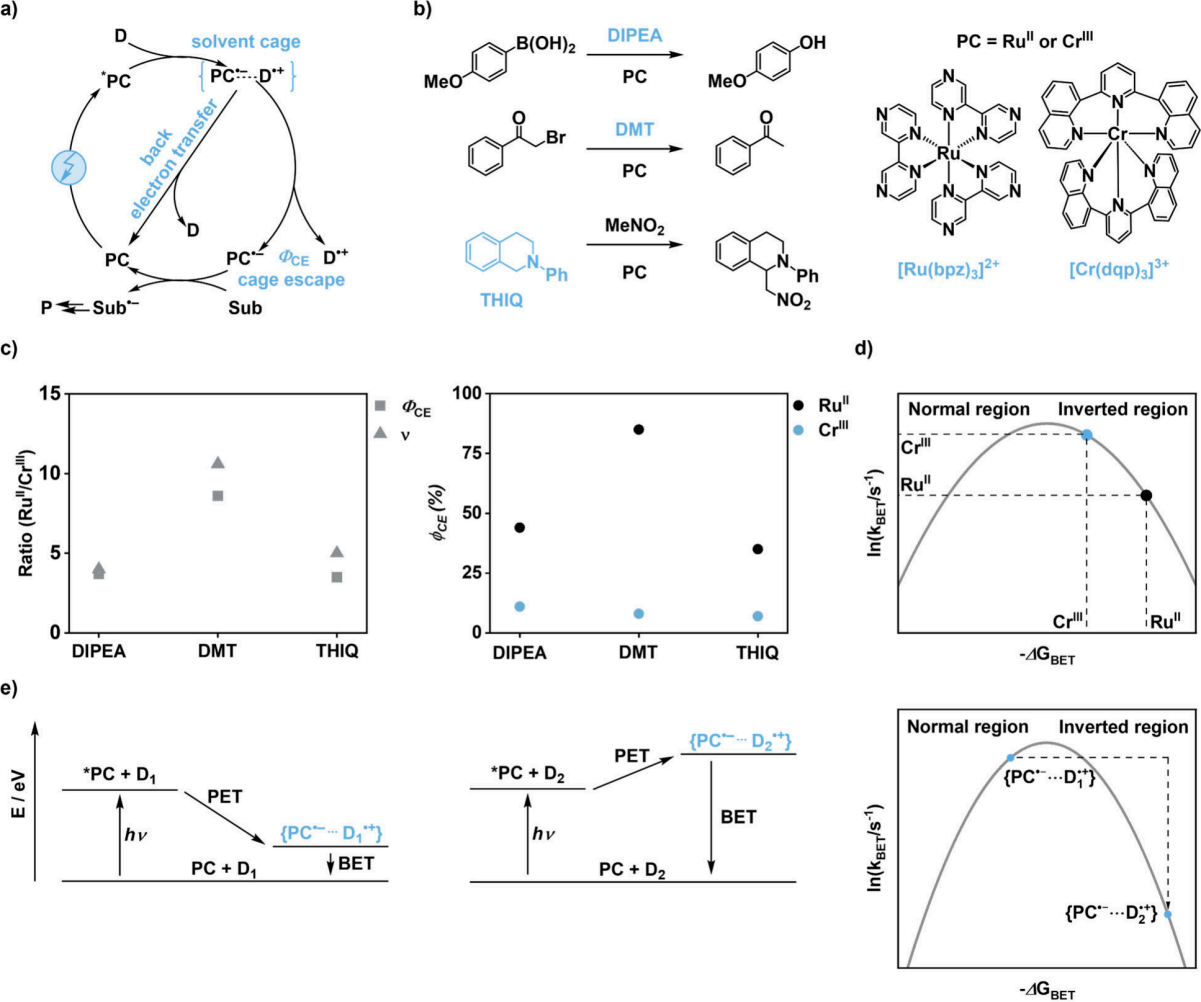
(a) Catalytic cycle of a photocatalyst (PC) undergoing a reductive quenching mechanism with an electron donor (D). Upon excitation, the photocatalyst undergoes PET, resulting in a reduced photocatalyst and an oxidized donor, both initially confined within a solvent cage. Spontaneous back electron transfer (BET) can occur before the geminate radical pair escapes from the solvent cage. Productive photoredox chemistry is only possible if cage escape succeeds, allowing the reduced photocatalyst to transfer an electron to the substrate (Sub), which then undergoes subsequent reaction steps to form the desired product (P). (b) Three different photochemical reactions involving three distinct electron donors investigated in the study (DIPEA = *N*,*N*-diisopropylethylamine, DMT = *N*,*N*-dimethyl-*p*-toluidine, and THIQ = 2-phenyl-1,2,3,4-tetrahydroisoquinoline) along with molecular structure of employed [Ru­(bpz)_3_]^2+^ (bpz = 2,2′-bipyrazine) or [Cr­(dqp)_2_]^3+^ (dqp = 2,6-di­(quinoline-8-yl)­pyridine) photocatalysts. (c) Ratios of cage escape quantum yields (ϕ_CE_) and initial product formation rates (ν) comparing scenarios involving either a Ru^II^ or a Cr^III^ photocatalyst along with cage escape quantum yields for electron donors investigated.^3^ (d) Marcus parabola illustrating how the rate constant for back electron transfer (*k*_BET_) within the solvent cage depends on its driving force (Δ*G*_BET_).^3^ For a given electron donor, in-cage back electron transfer involving the reduced Ru^II^ complex occurs further into the inverted regime and is therefore slower than involving the reduced Cr^III^ complex. (e) Energy level diagram for exergonic and endergonic initial PET illustrating that spontaneous back electron transfer (BET) within the solvent cage becomes more exergonic as the PET becomes increasingly endergonic.^35^ Marcus parabola depicts the expected effect on *k*_BET_ for the two scenarios.

We observed that in some cases the overall rates of photoredox product formation correlate with the cage escape quantum yields (Figure 3c).^3^ The aerobic hydroxylation of an aryl boronic acid, the reductive debromination of 2-bromoacetophenone, and an aza-Henry reaction (Figure 3b) all proceed faster with the ruthenium complex [Ru­(bpz)_3_]^2+^ than with the chromium complex [Cr­(dqp)_2_]^3+^ by factors that correspond quite closely to the differences in cage escape quantum yields between these two photocatalysts (bpz = 2,2′-bipyrazine, dqp = 2,6-di­(quinoline-8-yl)­pyridine). Luminescence quenching experiments indicate that the PET reactions are equally fast under the conditions used for these benchmark photoreactions,^36^ but cage escape quantum yields are systematically much higher for the Ru^II^ complex than for the Cr^III^ complex across different electron donors investigated (Figure 3c).^3^ Remarkably, these differences in cage escape quantum yield have a decisive influence on the overall product formation rate and the overall quantum yield. The latter is expected to be the product of the individual quantum yields of all elementary reaction steps on the path from the starting material to the final product.^37^ The observation that the cage escape quantum yield can be decisive is therefore important. Similar observations have also been made in independent studies.^38^ This clearly shows that luminescence quenching alone is an insufficient descriptor of productive photoredox reactivity.

The physical origin of the systematically higher cage escape quantum yield for the Ru^II^ complex compared to the Cr^III^ complex is not entirely clear. While our original paper primarily discusses driving force effects, we have also considered the possibility of spin effects,^3^ similar to cage escape studies with other systems.^38,39^ Driving force effects were already the focus of early studies and are based on the idea that back electron transfer reactions between solvent-caged radical pairs are often so exothermic that they lie in the inverted region of Marcus theory.^40^ In this region, electron transfer rates decrease with increasing driving force. For the Ru^II^ photocatalyst, the driving force for back electron transfer inside the cage is systematically higher than for the Cr^III^ photocatalyst (Figure 3d). This could explain the higher cage escape quantum yield for the ruthenium complex compared to the chromium complex.

Within this interpretative framework, there is likely a delicate balance between the initial PET and the back electron transfer within the solvent cage that critically influences cage escape, overall reaction rates, and quantum yields (Figure 3e). The underlying reasoning is that strongly exergonic PET generates low-energy radical pairs, for which back electron transfer may occur near the activationless region of Marcus theory, potentially leading to faster recombination than in weakly exergonic or endergonic PET reactions, which produce higher-energy primary radical pairs whose back electron transfer may fall within the Marcus inverted region and thus proceed more slowly. Recent work on new types of photoactive Cr^III^ complexes has provided indirect evidence supporting this hypothesis,^35^ although further investigation is needed to confirm or refute it. If the photocatalyst has a sufficiently long excited-state lifetime, the initial PET can be endergonic by up to 0.5 eV or more. This may not only help maximize the conversion of light energy into chemical energy, but also improve cage escape quantum yields and reaction quantum yields in photoredox catalysis.

Many photoredox studies tend to focus on optimizing excited-state quenching by substrates, often by introducing relatively high driving forces for PET. While an efficient initial photoreaction is important, the subsequent light-independent step of cage escape appears to follow a different set of rules, some of which may conflict with a high driving force for PET.

We conclude this section by noting that considerations of solvent-cage dynamics introduce a new, controllable design element beyond the initial photoexcitation step, enabling higher overall quantum yields and more efficient photoredox reactions.^41^

## Picosecond Photoreactivity from Excited Organic Radical Ions

Electronically excited organic radical ions began to attract increasing attention from the synthetic organic chemistry community around 2014 with the emergence of the consecutive photoinduced electron transfer process known as ConPET and the development of synthetic molecular photoelectrochemistry,^42^ also referred to as electron primed photocatalysis.^43–45^ Common organic radical ions typically have excited state lifetimes of only a few picoseconds,^46,47^ which are generally considered too short to allow for diffusion based bimolecular reactions.^48,49^ Moreover, these species are chemically unstable and prone to further reactions.^50,51^ For example, radical anions can undergo protonation and additional one electron reduction, leading to closed shell two electron reduction products that exhibit nanosecond excited state lifetimes and may themselves be photoactive.^18,52–54^ In addition, excitation of radical anions can yield highly reducing solvated electrons via a process referred to as photodetachment or photoionization.^18,55^ These two characteristics, the ultrashort lifetimes and inherent chemical instability, have sparked significant debate regarding whether excited radical ions are indeed the true catalytic species as many synthetic studies claim, or whether the observed photochemistry is actually driven by degradation products or solvated electrons (Figure 4a).^18,55–58^

**Figure 4:**
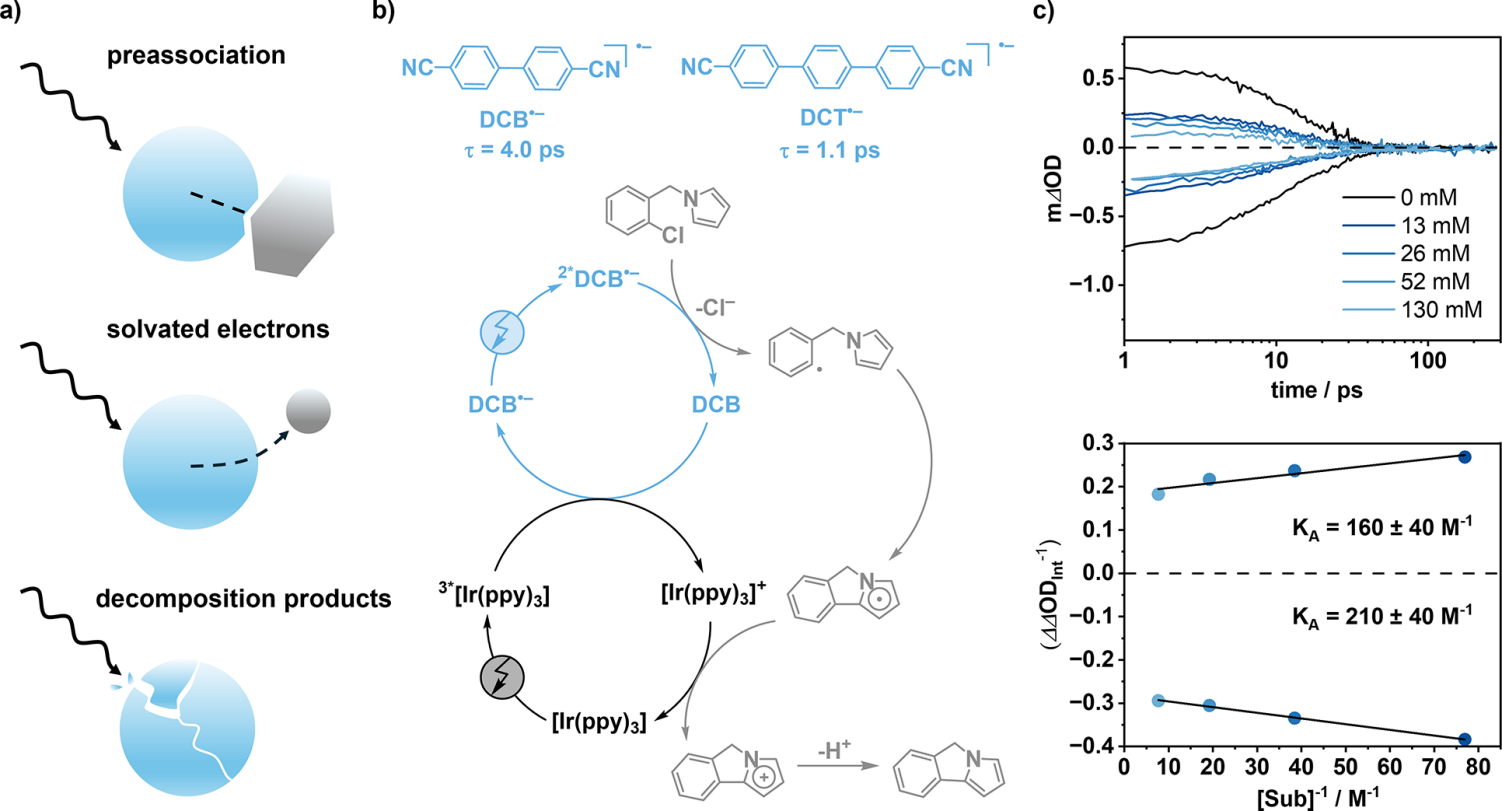
(a) Conceptual illustration of possible photochemical pathways upon excitation of radical ions: Preassociation of radical ion and substrate followed by PET, ejection of highly reducing solvated electrons upon irradiation, and photodecomposition of radical ions to yield long-lived (photo)­redox active byproducts. (b) Molecular structures of the radical anion forms of DCT and DCB, along with the structure of a model substrate used for reductive dehalogenation followed by intramolecular trapping of the resulting aryl radical by an *N*-alkylated pyrrole unit to form a cyclized product. The DCB radical anion was generated using a ConPET mechanism involving tris­(2-phenylpyridine)iridium ([Ir­(ppy)_3_]) as cocatalyst.^2^ (c) Decay of ^2*^DCB^•–^ monitored by ground-state bleach recovery (lower part) and excited-state absorption decay (upper part) in the presence of increasing concentrations of the model substrate, along with the corresponding Benesi–Hildebrand plot.^2^

Against this background, we explored the possibility that the radical anions of 4,4″-dicyano-*p*-terphenyl (DCT) and 4,4′-dicyanobiphenyl (DCB) form aggregates in solution with aryl halogenide substrate molecules and searched for direct spectroscopic evidence of PET within these aggregates on the picosecond time scale.^2^ Transient ultraviolet visible absorption spectroscopy provided evidence for quenching of the excited state of DCB^•–^ in the presence of an aryl chloride reaction partner. We interpreted this as a direct reaction occurring within the aggregated radical anion and substrate pair, held together by an association constant of approximately 200 M^–1^ Figure 4c). This quenching of a radical anion excited state, indicated by reduced signal amplitudes with largely unchanged dynamics (Figure 4c), complements a rare earlier report in which the lifetime of a radical cation excited state was observed to decrease on the picosecond time scale.^59^

Together, these two radical ion-substrate pairs provide perhaps the clearest current evidence for direct radical ion photoreactivity relevant to modern photoredox catalysis.^50^ Moreover, the study of the DCT and DCB radical anions revealed clear functional limits of this type of reactivity, including aspects related to (photo)­degradation, quantum yields, and the required irradiation conditions.^2^

First and foremost, direct radical anion reactivity was only observed in an overall redox-neutral reaction (Figure 4b) and in the absence of tertiary amine electron donors. These amines readily release a proton and a second electron following their initial one-electron oxidation,^60^ which likely promotes the formation of downstream products from the radical anions, such as the previously mentioned two-electron reduced, singly protonated closed-shell species that may themselves be photoactive.^18^ Second, the direct radical anion reactivity of DCB^•–^ with the aryl chloride reaction partner (Figure 4b) displayed a very modest product formation quantum yield of 0.005, even under high excitation power densities of approximately 100 mW/cm^2^. Third, prolonged irradiation for 3 h resulted in a significant decline in product formation quantum yield, even under optimized conditions. Taken together, these observations indicate that direct radical anion photoreactivity can indeed occur and may plausibly represent a contributing pathway at the onset of a photoredox reaction. However, it is relatively inefficient, and over time, degradation of the radical anions into other species such as photoactive reduction products or solvated electrons can become increasingly important,^2^ ultimately emerging as the dominant drivers of the photoredox process.^18,50^

In summary, while excited radical ions enable extreme-redox transformations, identifying the operative mechanisms remains essential for improving reaction predictability and selectivity. Recognizing preassociation as a viable pathway for productive PET opens new opportunities that may inform future photocatalyst development by introducing additional, interaction-based design elements capable of enabling photochemistry beyond the diffusion limit. Recent work proposes a unified view of radical anion photoredox chemistry, in which the radical anion either reacts with electrophiles to form a super-reducing photoreagent or generates solvated electrons that act as the dominant drivers of the photoreactivity.^18^

## Anti-Kasha Behavior

Kasha’s rule states that only the lowest electronically excited state of a given spin multiplicity exhibits luminescence.^61^ In closed-shell molecules, this corresponds to fluorescence from the S_1_ state and phosphorescence from the T_1_ state.^60^ All higher excited states (S_2_, S_3_, T_2_, T_3_, etc.) relax too rapidly to these lowest states for luminescence to occur. Early photochemists observed that both luminescence and photochemistry typically proceed from the lowest excited state of a given multiplicity.^62^ However, exceptions exist in which photochemical reactions originate from higher excited states, usually photodissociation reactions or intramolecular processes such as photoisomerizations.^63,64^ These cases are often said to break Kasha’s rule or exhibit anti-Kasha behavior. Some have argued that “the seminal definition by Kasha must remain unchanged for the sake of clarity”, suggesting the rule should not be applied to photochemistry.^65^ Nevertheless, references to “Kasha rule violations” or “anti-Kasha behavior” have become common in photochemical literature, and many researchers today consider this usage meaningful, given the parallels in excited-state behavior that govern both luminescence and photochemical reactivity.

Kasha’s rule is almost always the starting point for designing photochemical reactions, because one usually implicitly assumes that productive photochemical reactivity arises only from the lowest excited state. In photoredox catalysis, the initial light-dependent step is typically PET, and the redox potential for this step is estimated using the ground-state redox potentials together with the energy of the lowest excited state.^66^ Similarly, energy transfer reactions are usually designed based on the energy of the lowest triplet excited state.^67^ Only in rare and well-documented exceptions to Kasha’s rule, such as zinc­(II) porphyrins, are higher excited states like S_2_ also taken into account.^68–71^

Modern photoredox catalysis research has repeatedly invoked anti-Kasha behavior in cases where shorter-wavelength light induces a chemical reaction that is not observed under longer-wavelength irradiation.^72,73^ While such results may suggest reactivity from higher excited states, wavelength-dependent behavior often arises from more trivial causes, such as the formation of photoactive degradation products or formation of solvated electrons.^18,50,55,74^ In bimolecular reactions, claims of anti-Kasha behavior frequently overlook that internal conversion, with few exceptions such as the azulene family,^75^ typically occurs on a pico- or subpicosecond time scale. At such rates, diffusion-controlled encounters are essentially excluded, and only catalyst-substrate complexes that are preassociated prior to excitation may react before relaxation.^50^ As a result, direct evidence for bimolecular anti-Kasha reactivity remains exceedingly rare.

Against this background, and building on the insights into radical anion-substrate aggregation and the excited-state photoreactivity of organic radical anions discussed in the previous section, we set out to investigate the potential for bimolecular anti-Kasha reactivity of the DCT radical anion.^1^ This open-shell species has a doublet ground state (D_0_), with the D_1_ and D_2_ excited states separated by approximately 1 eV (Figure 5a).^47^ This energetic spacing is reminiscent of the situation in azulenes, where the S_1_ and S_2_ states are also energetically well separated.^76^

**Figure 5:**
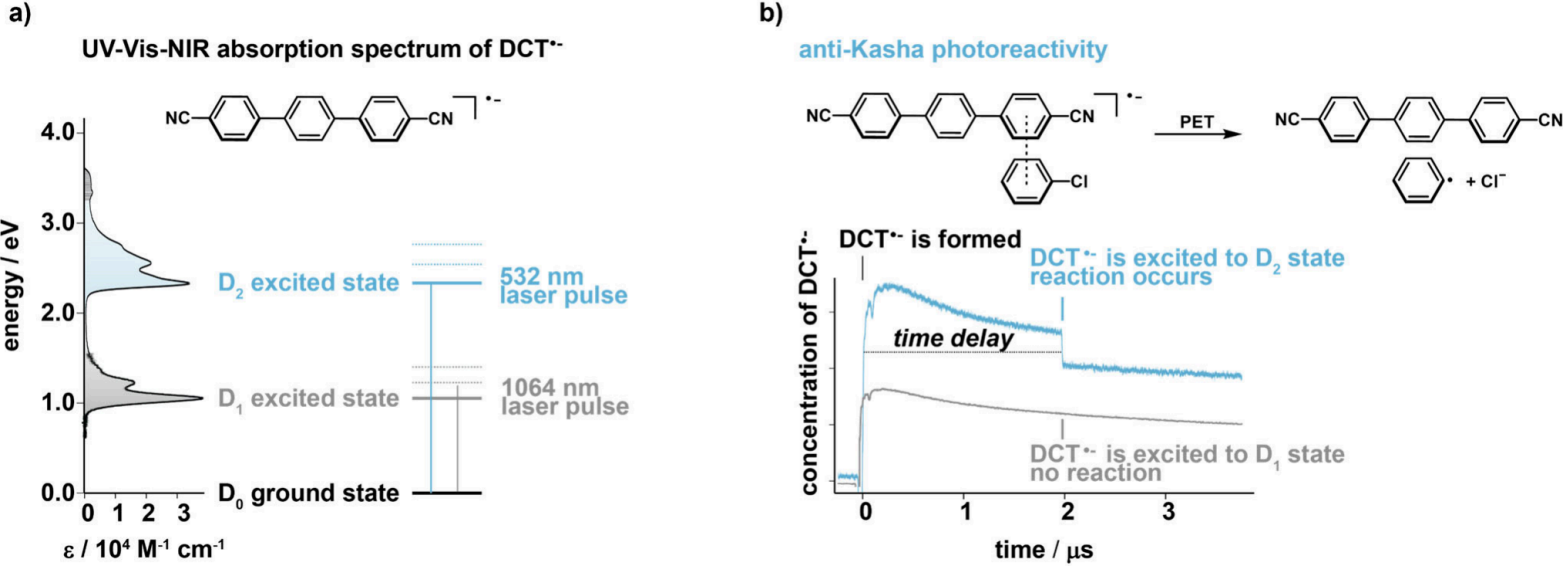
(a) Molecular structure of 4,4″-dicyano-*p*-terphenyl (DCT) and UV–vis–NIR absorption spectrum of the DCT radical anion in *N*,*N*-dimethylformamide, along with an energy level scheme showing the electronic ground state and the first and second electronically excited states, including schematic vibrational levels. (b) Top: Preassociation between the DCT radical anion and chlorobenzene and the PET reaction resulting in neutral DCT and one-electron reduced chlorobenzene, which leads to the release of an aryl radical and a chloride anion. Bottom: Results from pump–pump–probe experiments in which DCT^•–^ is formed by excitation of DCT in the presence of excess *N*,*N*-dimethylaniline in DMF at time zero, followed by spontaneous decay of DCT^•–^. After a time delay of 2 μs, a 532 nm laser pulse excites DCT^•–^ into the D_2_ state, inducing the photoreaction shown above, whereas excitation into the D_1_ state at 1064 nm does not lead to a detectable reaction.

Since DCT^•–^ is an unstable species, it was generated photochemically from DCT by using 355 nm laser pulses. These pulses promote the charge-neutral precursor compound into an electronically excited state in which it becomes strongly oxidizing. In this state, it can abstract an electron from an electron donor present in excess, such as *N,N*-dimethylaniline (DMA). The formation of DCT^•–^ was monitored at 500 nm, a wavelength where neither DCT nor any other reaction components absorb. This results in a more or less instantaneous buildup of the DCT^•–^ population upon the arrival of the 355 nm flash at time zero (Figure 5b). The DCT^•–^ population then begins to decay spontaneously due to its chemical instability. However, if followed by a second laser pulse on the microsecond time scale, DCT^•–^ can be excited and brought into a chemical reaction. After a time delay of 2 μs, DCT^•–^ was promoted either to its first excited state (D_1_) using a 1064 nm laser pulse or to its second excited state (D_2_) using a 532 nm laser pulse. When this pump–pump–probe experiment was performed in the presence of an excess electron acceptor such as chlorobenzene, a clear difference emerged between the two excitation wavelengths. Excitation to the D_2_ state led to an immediate depletion of the DCT^•–^ population, whereas D_1_ excitation had little to no effect. This depletion is interpreted as PET between preassociated DCT^•–^ and chlorobenzene, resulting in DCT and the chlorobenzene radical anion. Both of these species do not absorb at the detection wavelength of 500 nm. Evidently, this reaction occurs only from the D_2_ state and not from the D_1_ state, indicating a violation of Kasha’s rule.^1^

Systematic studies with ten different electron acceptors, specifically halogenated benzenes, provided insight into the driving force behind the anti-Kasha reactivity observed with D_2_-excited DCT^•–^. The key finding was that, aside from the necessity for preassociation due to the very short lifetime of the D_2_ excited state,^2,50^ a threshold driving force for PET of approximately 1.2 eV was required.^1^ This requirement likely reflects the fact that for the PET elementary step to proceed on a (sub)­picosecond time scale, the overall reaction system must be optimized so that nearly barrierless electron transfer can occur. This point is usually reached when the driving force equals the reorganization energy, λ. Reorganization energy values between 0.8 and 1.2 eV are common in molecular systems featuring PET,^77,78^ and in our system, it appears to be closer to the upper end of this range. This work follows a previous spectroscopic study that proposed higher excited-state reactivity from a radical cation with mesitylene.^59^

Anti-Kasha reactivity may be significantly more common than previously assumed. Emerging evidence in recent literature from both experimental and computational studies indicates that higher excited states can in fact be exploited in photophysical and photochemical processes.^79,80^ Given the aforementioned photochemical and chemical instability of radical ions, this class of compounds is unlikely to be the primary candidate for the systematic future implementation of anti-Kasha reactivity. In particular, wavelength-dependent reactivity in such systems may also arise from photodetachment processes leading to solvated electron formation rather than from intrinsic excited-state chemistry,^18,55^ similar to observations reported for transition metal complexes.^81–83^ We believe that anti-Kasha reactivity will become more systematically accessible through transition metal complexes, especially once targeted efforts are made to explore it.^84–88^

We conclude that despite continued debate over the underlying mechanisms, elucidating wavelength-dependent reactivity is essential for further progressing the field of photoredox catalysis, as it unlocks access to otherwise inaccessible thermodynamic driving forces and thereby expands the chemical toolbox for designing fundamentally new photochemical transformations.

## Concluding Remarks

Many synthetic photoredox studies have proposed reaction mechanisms involving multiple electronic excitations per catalytic turnover.^14^ Mechanistic investigations have confirmed that such processes are indeed viable, while also establishing clear boundary conditions under which they can realistically contribute to catalysis. For example, our work, along with studies by other researchers, shows that photochemically generated radical ions can undergo a secondary excitation through absorption of an additional photon and participate in direct photochemical reactions,^2,48,50^ as proposed by synthetic researchers.^42^ However, we also find that this is only possible after preassociation with substrates and occurs with low product formation quantum yield.^2,50^ Prolonged irradiation may generate two-electron reduced, singly protonated species responsible for driving the photochemistry.^52^ These species can themselves be more photoactive, which is consistent with observations reported by other research groups.^18,52,53,89,90^ In addition to these two-electron reduced photoproducts, solvated electrons are increasingly recognized as important catalytic species,^18,55,56,91^ as demonstrated in our earlier mechanistic studies and those of others.^81,83,92^

There is considerable opportunity for innovation in the design of photochemical reaction mechanisms, and photon upconversion is emerging as an attractive strategy in photocatalysis.^15,93^ Upconversion into the ultraviolet region is of particular interest, as it could extend the thermodynamic limits of photochemical reactivity.^21^ In this context, the issue of quantum yield becomes especially important.^37,94^ This factor warrants close attention for the practical application of photocatalysis, not only in relation to upconversion but more broadly.^95^ This is underscored by the fundamental finding that cage escape quantum yields play a decisive role in determining both reaction rates and overall quantum efficiencies in photoredox processes.^3^ The phenomenon of cage escape discussed here is conceptually related to radical rebound mechanisms, for which there is increasing experimental evidence, particularly in nickel-based cross-coupling reactions conducted under light irradiation.^96,97^

From a conceptual mechanistic standpoint, one of the most promising directions for future research may be the systematic design of (bimolecular) photoreactivity from higher excited states. A proof of principle has now been established, and the extent to which the concept of anti-Kasha reactivity can be systematically integrated into photochemistry remains an open and compelling question.^1^

The conclusions and perspectives outlined in the preceding sections largely stem from the cross-fertilization between photochemical research carried out by synthetic organic chemists and work by physical and inorganic chemists focused on time-resolved spectroscopy and mechanistic insight.^98^ This interdisciplinary interplay appears to be essential for the continued advancement of the field.^99^
